# Eine Neuordnung der Zeit? Zum Verhältnis von Zeitlichkeit, Kapitalismus und Staat im Zeichen der Pandemie

**DOI:** 10.1007/s11609-022-00466-w

**Published:** 2022-03-15

**Authors:** Lisa Suckert

**Affiliations:** Max-Planck-Institut für Gesellschaftsforschung, Paulstraße 3, 50676 Köln, Deutschland

**Keywords:** Coronapandemie, Kapitalismus, Krise, Staat, Zeitlichkeit, Zukunft, COVID-19 pandemic, Capitalism, Crisis, State, Temporality, Future, Pandémie de coronavirus, Capitalisme, Crise, État, Temporalité, Avenir

## Abstract

Ermöglicht die Coronapandemie eine Abkehr vom kapitalistischen Zeitregime? Bedingt die Krise eine nachhaltige Neuordnung der Zeit? Der Beitrag betrachtet die COVID-19-Pandemie und die mit ihr einhergehenden staatlichen Maßnahmen aus einer zeit- und wirtschaftssoziologischen Perspektive. Er macht deutlich, dass sich die sozialen und ökonomischen Verwerfungen der Pandemie auch als Ergebnis einer Kollision entgegengesetzter zeitlicher Logiken verstehen lassen. Die staatlichen Maßnahmen zur Pandemiebekämpfung erzwangen zunächst einen Umgang mit Zeit, der dem kapitalistischen Zeitregime und dessen Prinzipien – Kommodifizierung und rationale Verwertung von Zeit, Beschleunigung sowie Aneignung der Zukunft – widerspricht. Anders als dies z.B. Hartmut Rosa erhofft, impliziert diese „Rückkehr des Staates“ als zeitpolitische Ordnungsmacht jedoch noch keinen Pfadwechsel hin zu einer andauernden „Neuordnung der Zeit“. Der Beitrag zeigt, dass insbesondere jene staatlichen Interventionen, die über die reine Pandemiebekämpfung hinausgehen, als Vermittlungsversuche zwischen unterschiedlichen zeitlichen Logiken zu verstehen sind. Sie federn die Kollision entgegengesetzter zeitlicher Logiken zwar ab, erleichtern im Kern jedoch ein „Zurück“ zum kapitalistischen Zeitregime. Zeitbezogene Ungleichheiten werden dabei unbeirrt fortgeschrieben.

## Abstract (en)

Does the corona pandemic allow for a departure from the capitalist time regime? Can the crisis induce a more sustainable order of time? This article looks at the COVID-19 pandemic and the state interventions in response to it from the perspective of sociology of time and economic sociology. It shows that the social and economic disruptions attributed to the pandemic can be understood as the result of a collision of opposing temporal logics. In order to fight the pandemic, the state initially enforced ways of dealing with time that contradict the capitalist time regime and its major principles—commodification and rational use of time, acceleration, as well as appropriation of the future. However, and contrary to what e.g. Hartmut Rosa hopes, this “return of the state” as a time-political ordering power does not yet imply a path change towards a “new order of time” that lasts beyond the current state of emergency. The article shows that especially those state interventions that go beyond mere pandemic control can often be understood as attempts to mediate between different temporal logics. While they cushion the collision of opposing temporal logics, they are essentially intended to facilitate a “return” to the capitalist time regime. In doing so, they perpetuate time-related inequalities.

## Résumé

La pandémie de coronavirus permet-elle une sortie du régime temporel capitaliste ? La crise entraîne-t-elle la mise en place d’un nouvel ordre temporel durable ? Cet article se penche sur la pandémie de Covid-19 et les mesures étatiques auxquelles elle a donné lieu dans une perspective combinant sociologie du temps et sociologie économique. Il met en évidence le fait que les bouleversements sociaux et économiques causés par la pandémie peuvent être également conçus comme le résultat d’une collision entre des logiques temporelles opposées. Les mesures étatiques pour lutter contre la pandémie ont tout d’abord imposé un rapport au temps allant à l’encontre du régime temporel capitaliste et de ses principes (marchandisation et exploitation rationnelle du temps, accélération et appropriation de l’avenir). Contrairement à ce qu’espérait p. ex. Hartmut Rosa, ce « retour de l’État » comme puissance temporelle régulatrice n’implique toutefois pas un changement de trajectoire vers un « nouvel ordre temporel » durable. Cet article montre que notamment les interventions étatiques qui dépassent la simple lutte contre la pandémie doivent être conçues comme des tentatives de médiation entre différentes logiques temporelles. Elles amortissent certes la collision entre différentes logiques temporelles, facilitent cependant au fond un « retour » au régime temporel capitaliste. Ce faisant, les inégalités liées au temps sont imperturbablement prolongées.

## Einleitung

Bietet die Coronapandemie die Chance einer nachhaltigen Neuordnung der Zeit? Lässt sich die Krise als Wendepunkt verstehen, der eine veritable Abkehr von den Imperativen des kapitalistischen Zeitregimes erlaubt? Hartmut Rosa (2020) hat auf diese Fragen eine durchaus optimistische Antwort gegeben. Er beschreibt die Maßnahmen zur Pandemiebekämpfung – freilich noch unter dem Eindruck der ersten Welle – als staatlich forcierte „Zwangsentschleunigung“ (ebd., S. 195), als historischen Bruch mit dem omnipräsenten Modus der Steigerung. Die kollektive Erkenntnis, dass kapitalistische Beschleunigungsimperative durch einen handlungsfähigen Staat außer Kraft gesetzt werden können, begreift Rosa als „eine spektakuläre politische Selbstwirksamkeitserfahrung für die Gesellschaft“ (ebd., S. 199), die, so hofft er, die Chance eines nachhaltigen Pfadwechsels bietet.

Der nachfolgende Beitrag setzt sich kritisch mit Rosas These auseinander. Er nimmt sie zum Anlass, die Pandemie und die staatlichen Maßnahmen zu deren Eindämmung dezidiert aus einer zeitsoziologischen Perspektive zu betrachten. Inwiefern hat COVID-19 tatsächlich einen Umgang mit Zeit erforderlich gemacht, der sich von jenem kapitalistischen Zeitregime unterscheidet, das Wirtschaft und Gesellschaft für gewöhnlich prägt – und das neben Beschleunigungsimperativen auch durch die Kommodifizierung und rationale Verwertung von Zeit sowie einer kontinuierlichen Aneignung der Zukunft gekennzeichnet ist? Und: Welche Rolle spielt der Staat bei der Durchsetzung einer etwaigen Neuordnung der Zeit?

Meine Analyse, die sich auf zeit- und wirtschaftssoziologische Erkenntnisse stützt und diese mit empirischen Beispielen kontrastiert, bestätigt Rosas Befunde zunächst in Teilen. Erstens lassen sich die von der Pandemie ausgelösten sozialen und ökonomischen Verwerfungen auch als Ergebnis einer *Kollision entgegengesetzter zeitlicher Logiken* verstehen. Zeitliche Erfordernisse der Pandemiebekämpfung, wie das Verlangsamen vieler Aktivitäten, das (im ökonomischen Sinne) unproduktive Verstreichenlassen von Zeit oder die Hinfälligkeit von Zukunftsplänen widerstreben den Grundprinzipien des kapitalistischen Zeitregimes. Im Ausnahmezustand der Krise scheint die kapitalistische Zeitordnung erschüttert. Zweitens lässt sich dabei ein *Wiedererstarken des Staates als zeitpolitische Ordnungsmacht* erkennen. Staatliches Handeln greift aktiver in die zeitliche Ordnung von Wirtschaft und Gesellschaft ein und überlässt Fragen von Zeitnutzung, Beschleunigung, Synchronisation oder Zukunftsfähigkeit seltener als zuvor der „unsichtbaren Hand des Marktes“ oder der Selbstverwaltung von Organisationen.

Rosas These einer „spektakulären politischen Selbstwirksamkeitserfahrung für die Gesellschaft“ wird indes nicht bestätigt. Ich möchte im Verlauf des Beitrags zeigen, dass die pandemiepolitischen Eingriffe nicht pauschal als das staatliche Durchsetzen einer zeitlichen Logik *gegen* den Kapitalismus begriffen werden können, wie Rosa suggeriert (ebd., S. 201 f.). Dies gilt insbesondere für jene Interventionen, die nicht auf die Pandemiebekämpfung im engeren Sinne, sondern auf die Bewältigung der sozialen und ökonomischen Folgen abzielen. Sie lassen sich vielmehr als Versuch verstehen, zwischen verschiedenen zeitlichen Logiken zu vermitteln, und ermöglichen so die Aufrechterhaltung des kapitalistischen Zeitregimes über die Krise hinaus. Zudem verwischt der Befund eines „historisch beispiellosen Prozess[es] der Entschleunigung“ (ebd., S. 207; Hervorheb. weggel.), an dem Rosa trotz mancherlei Relativierung im Kern festhält, vielfältige Ambivalenzen und Ungleichheiten. Zwar hat die pandemiebedingte Erschütterung der zeitlichen Ordnung zahlreiche Bruchstellen des kapitalistischen Zeitregimes deutlicher sichtbar gemacht. Sie hat aber vor allem – und dies nicht zuletzt durch staatliche Eingriffe – zeitbezogene Ungleichheiten und Nöte fortgeschrieben und verschärft. Eine (zeitweilige) Entschleunigung im Sinne einer Befreiung aus den Zwängen einer kapitalistischen Zeitordnung war wenigen, ohnehin schon privilegierten Milieus vorbehalten. Eine Verstetigung dieser „Neuordnung der Zeit“ über den Ausnahmezustand hinaus erscheint daher wenig wahrscheinlich – und nur bedingt wünschenswert.

Der Beitrag entwickelt dieses Argument in vier Schritten. Der nachfolgende Abschnitt 2 arbeitet die drei zentralen Charakteristika des kapitalistischen Zeitregimes heraus: Kommodifizierung und rationale Verwertung von Zeit, Beschleunigung sowie Appropriation der Zukunft. Abschnitt 3 zeigt auf, wie dieses Zeitregime im Zusammenspiel von Selbstkontrolle und staatlicher Rahmung, Märkten und Organisationen durchgesetzt wird. Aufbauend auf dieser Rekapitulation der zeitlichen Ordnung von Wirtschaft und Gesellschaft „in Normalzeiten“ widmen sich die darauffolgenden beiden Abschnitte den durch die Pandemie ausgelösten Umbrüchen. Abschnitt 4 stellt die Krise als Kollision der unterschiedlichen zeitlichen Logiken von Pandemiebekämpfung und Kapitalismus dar. So zeichnen sich in der Pandemie neue Imperative von Verlangsamung und Geduld ab, wir erleben eine Redistribution und Dekommodifizierung von Zeitbudgets, und die Zukunft wird zu einer Sphäre radikaler Unsicherheit – all dies betrifft verschiedene Bevölkerungsgruppen jedoch in unterschiedlicher Weise. Abschnitt 5 betrachtet schließlich den über die Maßnahmen zur direkten Pandemiebekämpfung hinausgehenden zweiten Typus pandemiepolitischer Interventionen, der auf das Abfedern der wirtschaftlichen und gesellschaftlichen Krisenfolgen zielt. Aus einer zeitsoziologischen Perspektive wird deutlich, dass diese Maßnahmen verstärkt in die zeitliche Ordnung von Wirtschaft und Gesellschaft eingreifen, um zwischen den unterschiedlichen zeitlichen Logiken zu vermitteln. Die abschließende Diskussion reflektiert die Potenziale und Risiken dieser Neuordnung der Zeit, macht aber vor allem deutlich, dass es zum Abbau zeitbezogener Ungleichheiten mehr bedarf als einer bloßen „Rückkehr des Staates“ als zeitpolitische Ordnungsmacht.

## Die zeitliche Ordnung des Kapitalismus

Aus zeitsoziologischer Perspektive stellt der Kapitalismus nicht nur ein Produktionsregime dar, sondern geht mit einer spezifischen zeitlichen Ordnung einher. Diese bedingt die Wahrnehmung und Nutzung von Zeit weit über die Sphäre der Wirtschaft hinaus (Jessop 2007, S. 178 ff.; Sewell Jr 2008). Wie aber lässt sich die zeitliche Ordnung beschreiben, die kapitalistische Gesellschaften prägt? Im Folgenden möchte ich, im Rückgriff auf die zeitsoziologische Literatur (vgl. insbesondere Adam 2006a S. 123 ff.; Reckwitz 2016), drei Merkmale herausarbeiten, die das kapitalistische Zeitregime wesentlich charakterisieren (vgl. Suckert 2021). Die Rede von *dem* kapitalistischen Zeitregime ist dabei selbstredend als analytischer Idealtypus zu verstehen. Genauso wie *der* Kapitalismus in ganz unterschiedlichen nationalen Spielarten und historischen Ausprägungen existiert (Boltanski und Chiapello 2007; Hall und Soskice 2001), variieren auch die entsprechenden zeitlichen Regime und deren institutionelle Verankerung. Gleichwohl zeichnen sich Wirtschafts- und Gesellschaftsordnungen, die durch Lohnarbeit, Wettbewerb und Profitorientierung geprägt sind, – in Normalzeiten – durch die Kombination der nachfolgend dargestellten drei Merkmale aus.^1^

### Kommodifizierung und expansive Verwertung von Zeit

Wenngleich Soziolog*innen wie Barbara Adam (2005, S. 23 ff., 2006b) oder Pierre Bourdieu (2000) die De-Kontextualisierung und Anonymisierung von Zeit immer wieder kritisiert haben, sind sie doch ein wesentliches Charakteristikum der Moderne. Nur wenn Zeit eine standardisierte und universelle, das heißt über die Grenzen von Organisationen, Feldern oder Systemen hinweg gültige und messbare Größe ist, können moderne Gesellschaften ihre unterschiedlichen Rhythmen synchronisieren und funktionsfähig bleiben (vgl. Nassehi 2008; aber auch bereits Durkheim 2001, S. 11 f.; Elias 1984 oder Sorokin und Merton 1937). Die Abstraktion von Zeit ist dabei gleichzeitig eine wesentliche Voraussetzung des Kapitalismus. Sie erlaubt es, Zeit mit dem abstraktesten aller Medien gleichzusetzen: dem Geld (Esposito 2011; Simmel 1989, S. 706 f.). Zeit lässt sich nur mit einem Preis versehen und als Ware handeln, wenn sie als von ihrem Träger losgelöste Einheit denkbar wird. Zentrale kapitalistische Institutionen, wie Lohnarbeit oder Zinsen, sind auf diese Kommodifizierung und damit die Abstraktion von Zeit angewiesen.

Als Ware muss Zeit nun – wie jede andere Ressource im Kapitalismus – gewinnbringend genutzt werden. „*Zeitvergeudung* ist also die erste und prinzipiell schwerste aller Sünden“, folgert bereits Max Weber (2011, S. 167) in seiner Studie über den kapitalistischen Geist. Die Gleichsetzung mit Geld macht Zeit zu einem knappen Gut, dessen vollen Wert es zu extrahieren gilt. Zeit darf folglich nicht ungenutzt verstreichen, sondern muss zielgerichtet, das heißt im Sinne der Kapitalakkumulation investiert und verwertet werden. Die Kommodifizierung von Zeit schlägt sich daher in der Tendenz nieder, menschliche Zeit zunehmend auf jene Prozesse auszurichten, die diese Akkumulation im Kern antreiben: Produktion und Konsum.

Die Expansion dieser kapitalistischen Verwertung von Zeit stellt Karl Marx besonders prägnant in seinen Überlegungen zum Wesen des Arbeitstages dar (Marx 1984, S. 245 ff.). Er zeichnet historisch nach, wie Arbeitgeber versuchen, die für den Lohn zu erbringende Arbeitszeit sukzessive auszuweiten, etwa durch Schichtsysteme oder die Kürzung „überflüssiger“ Pausen. Die heutige Arbeitswelt unterscheidet sich zweifelsohne von jener, die Marx für die Mitte des 19. Jahrhunderts beschreibt: Die durchschnittliche Wochenarbeitszeit wurde in Deutschland bis in die 1990er-Jahre hinein stetig reduziert und mit dem Arbeitszeitgesetz von 1969 „in der Regel“ auf 40 Stunden festgeschrieben (vgl. auch Maurer 1992). Der grundlegende Konflikt besteht jedoch auch im Kapitalismus des 21. Jahrhunderts fort. Die Flexibilisierung von Arbeitszeiten hat seit Mitte der 1990er-Jahre nicht nur zu einem leichten Anstieg der durchschnittlichen Wochenarbeitszeit, sondern auch zu einer Entgrenzung des Arbeitstages beigetragen. In der flexiblen, globalen und digitalen Arbeitswelt der Gegenwart wird die Unterscheidung von Arbeitszeit und Freizeit zunehmend – und häufig durchaus bewusst – verwischt (Snyder 2016).^2^ Ebenso lassen sich Debatten um die Heraufsetzung des Renteneintrittsalters oder die Verkürzung der Schul- und Studienzeit als Versuche verstehen, die individuelle Lebensarbeitszeit zu erhöhen (Han und Moen 1999; Lynch 2012).

Darüber hinaus zeigt sich eine qualitative Umdeutung von Zeit. So diagnostiziert Helga Nowotny (1989, S. 115 ff.) eine fortschreitende Umwandlung von privater, nicht-monetarisierter Zeit in öffentliche, monetarisierte Zeit. Freizeit wird beispielsweise in kommerzialisierte Formen der Erholung überführt. Befeuert durch immer neue Angebote der Freizeit- und Unterhaltungsindustrie wird sie nur noch selten als freie Zeit verbracht, sondern stattdessen, oft gegen viel Geld, konsumiert. In ähnlicher Weise werden zeitaufwändige, aber vormals unvergütete Tätigkeiten wie Haushaltsführung oder die Sorge für Kinder und Alte in (gering)bezahlte Arbeitszeit transformiert (Bakker 2007; Fraser 2016). Der entsprechende Zeitaufwand lässt sich alsdann auf dem Markt anbieten oder nachfragen. Den Akteuren, die bislang mit derartigen Aufgaben betraut waren, insbesondere Frauen, eröffnet sich bei entsprechenden Ressourcen die Möglichkeit, sich „freizukaufen“ (Gupta 2007). Zeit, die bislang für Haus- und Sorgearbeit verloren ging, kann nun zurückgekauft werden, um sie in die eigene Erwerbsarbeit wertschöpfend zu investieren, die potenziell besser bezahlt ist, als jener Zeitaufwand, den etwa Betreuungspersonal und Putzhilfen nun erbringen. In der Folge ist ein immer größerer Anteil menschlicher Lebenszeit dem kapitalistischen Regime und seinen Imperativen ausgesetzt.

### Beschleunigung

Die Intensivierung der Zeitnutzung stellt ein weiteres Wesensmerkmal des kapitalistischen Zeitregimes dar. Bereits Georg Simmel (1989, S. 696 ff.) beschreibt die Beschleunigungstendenz als ein Spezifikum der Moderne – ein Befund, den etwa Reinhart Koselleck (1989) oder Hartmut Rosa (2013) untermauern. Der Kapitalismus begünstigt diese moderne Neigung, das Tempo zu erhöhen (vgl. Snyder 2013). Wo Zeit Geld kostet, kann es niemals schnell genug gehen. Die grundlegenden kapitalistischen Prinzipien von Wettbewerb und Wachstum treiben die Akteure dazu an, schneller zu sein als die Konkurrenz, nicht stehen zu bleiben oder sich mit dem Erreichten zufrieden zu geben. Joseph Schumpeters (2000) Konzept der „schöpferischen Zerstörung“, mit dem er das Wesen des Kapitalismus umschreibt, verweist auf ebendiese unaufhörliche Beschleunigung, das heißt eine Welt, die sich immer schneller im Umbruch befinden muss. Mark Zuckerberg hat Schumpeters Einsicht einschlägig für den Kapitalismus des 21. Jahrhunderts reformuliert: „Move fast and break things“.

In der Tat scheinen jene Prozesse, die das gegenwärtige kapitalistische Regime prägen, die Geschwindigkeit weiter zu erhöhen. So schläft in einer Welt des globalen Wettbewerbs die Konkurrenz nie, denn irgendwo auf dem Erdball wird immer produziert, konsumiert, noch schneller entwickelt. Gleichzeitig müssen sich viele Wirtschafts- und Lebensbereiche nach dem rasanten Tempo der Finanzmärkte ausrichten, wo Geld zum Zweck der Akkumulation immer schneller umgeschlagen wird. Zwar müsse der technologische Fortschritt, so betont etwa Judy Wajcman (2008, 2015), nicht per se beschleunigend wirken, sondern könnte auch dazu dienen, zeitliche Freiräume der Entschleunigung zu schaffen. Tatsächlich werden technische und vor allem digitale Innovationen im Kapitalismus jedoch überwiegend genutzt, um den Takt von Produktion, Kommerzialisierung und Konsum weiter zu erhöhen.

Beschleunigung wird so zu einem allgemeingültigen Anspruch, dem sich alle Mitglieder der Gesellschaft zu unterwerfen haben – den aber häufig jene am besten einlösen können, die über entsprechende Ressourcen verfügen (Sharma 2014). Dass die zunehmende Geschwindigkeit für eine Reihe gesellschaftlicher Probleme verantwortlich zeichnet, ist eine zentrale Einsicht der Zeitsoziologie, die nicht müde wird, der kapitalistischen Beschleunigungstendenz vielfältige psychische, soziale und politische Verschleißerscheinungen zuzuschreiben (Rosa 2016; Snyder 2016; Wajcman und Dodd 2016).

### Aneignung der Zukunft

Gegen diese kritische Perspektive lässt sich einwenden, dass gerade der Imperativ stetiger Beschleunigung die Anpassungsfähigkeit des Kapitalismus bedingt. Im Kapitalismus bieten sich für Akteure Anreize, es besser zu machen, schneller und effektiver zu werden, der Konkurrenz einen Schritt voraus zu sein. Akteure werden angehalten, Fortschritt zu befördern und ihr Handeln auf die Zukunft auszurichten.

In der Tat wurde der Kapitalismus in den letzten Jahren wiederholt als Wirtschaftsordnung beschrieben, die exzessiv auf die Zukunft ausgerichtet ist (Urry 2016; Wenzel et al. 2020; vgl. Beckert und Suckert 2021). Insbesondere Jens Beckert (2013, 2016) hat darauf verwiesen, dass wichtige Elemente kapitalistischer Dynamik, wie Kredite, Investitionen, Innovation oder Konsum von Zukunftsvorstellungen motiviert werden. Der Kapitalismus wird angetrieben vom Glauben der Akteure an eine offene Zukunft, von deren Möglichkeiten sich profitieren lässt.

Die zugrunde liegende Fixierung auf die Zukunft ist jedoch – wie für den Kapitalismus nicht unüblich (Honneth 2002) – zutiefst paradox. So ist der Kapitalismus einerseits auf die Vorstellung einer *offenen* Zukunft angewiesen; in modernen, kapitalistischen Gesellschaften erscheint die Zukunft eben nicht als von höheren Kräften (vorher)bestimmt. Unternehmertum erfordert eine Zukunft, die kein hinzunehmendes Schicksal ist, sondern eine wandelbare Sphäre, die sich ausgehend von der Gegenwart gestalten und nutzen lässt. Diese Aneignung der Zukunft, durch die Zukunft als Ressource zugänglich wird, erfordert jedoch andererseits ihr stetiges *Einhegen* und *Eingrenzen*. Um die Zukunft nutzbar zu machen, darf sie nicht determiniert, aber eben auch nicht willkürlich erscheinen, sondern muss den Anforderungen von Planbarkeit und Regularität unterworfen werden. Barbara Adam beschreibt diese Unterwerfung als eine „Kolonialisierung der Zukunft“ (Adam 2006a, S. 140 ff.).

Eine Vielzahl von kapitalistischen Alltagspraktiken hat ebendiese ambivalente Funktion: die Zukunft durch Pläne, Berechnungen, Simulationen und Prognosen gleichzeitig zu öffnen und zu schließen und dadurch gangbar zu machen. So zielen Businesspläne (Giraudeau 2018) oder Finanzkalkulationen, die die Zukunft „diskontieren“ (Doganova 2018; Muniesa et al. 2017), zwar einerseits darauf, alternative Zukünfte denkbar zu machen (Beckert 2021) – die Zukunft wird durch sie jedoch keinesfalls entfesselt, allenfalls „einen Spalt weit“ geöffnet. Sie wird, wie Klaus Kraemer (2021) betont, als antizipierbare Kausalkette zu einer beherrschbaren Größe umgedeutet, die man sich zu eigen machen kann. Der Kapitalismus, der maßgeblich durch die Zukunftsorientierung der Akteure angetrieben wird, erfordert und bedingt somit eine Zukunft, die zwar als offen und gestaltbar erscheint, dabei aber in erster Linie plan- und vorhersehbar sein muss.

## Die ordoliberale Durchsetzung des kapitalistischen Zeitregimes

Wie wird die zeitliche Ordnung des Kapitalismus in Wirtschaft und Gesellschaft durchgesetzt? Die Kommodifizierung und rationale Verwertung von Zeit, Beschleunigung und Aneignung der Zukunft werden – zumindest hierzulande und heutzutage (vgl. Thompson 1967) – nicht durch körperliche Gewalt erzwungen. Stattdessen zeigt sich ein Zusammenspiel von staatlicher Rahmung mit marktorientierter und organisationsinterner Koordination, das sich im Sinn eines „zeitbezogenen Ordoliberalismus“^3^ verstehen lässt.

Charles Tilly (1994) hat eindrücklich beschrieben, wie die Herausbildung von konsolidierten Nationalstaaten die Durchsetzung allgemeingültiger Zeitlichkeiten bedingt und erfordert. Die Zeit seiner Bürger*innen zu ordnen und zu kontrollieren, so Tilly, ist eine wesentliche Funktion nationalstaatlicher Herrschaft. Es lässt sich daher, auch jenseits des derzeitigen Ausnahmezustandes, kaum bezweifeln, dass staatliche Ge- und Verbote, Normen und institutionelle Anreize beeinflussen, auf welche Art und Weise Bürger*innnen über Zeit denken, sie nutzen und wahrnehmen (Manow 1998; Rice et al. 2006). So tritt der Staat beispielsweise als zeitliche Ordnungsmacht auf, wenn er das Renteneintrittsalter festlegt oder Kinderarbeit verbietet, die maximale wöchentliche Arbeitszeit reguliert oder regelmäßige Arbeitspausen festschreibt; wenn er Feiertage oder den Wechsel von Sommer- zu Winterzeit bestimmt, Ladenöffnungs- und Vertragslaufzeiten reglementiert; wenn er Ansprüche auf Kinderbetreuung, Elternzeit oder Urlaub gewährleistet; wenn er Nachtflugverbote und Sperrstunden verhängt oder Versicherungen und Rücklagen zur Absicherung gegen zukünftige Unwägbarkeiten verlangt.

In seiner Analyse konstatiert Tilly jedoch bereits in den 1990er-Jahren, dass der Zenit „staatlicher Zeiten“ überschritten sei (1994, S. 291 ff.). Der Machtverlust des Nationalstaats, der sich angesichts von Globalisierung und Finanzialisierung abzeichnet, würde auch die staatliche Fähigkeit beeinträchtigen, als zeitpolitische Ordnungsmacht aufzutreten. Der Staat, so Tilly, gibt zunehmend die Macht ab, die Zeit seiner Bürger*innen zu gestalten.

Für das Deutschland der 1990er-Jahre zeichnet Philip Manow (1998) in ähnlicher Weise eine Verschiebung hin zu einem liberalen und damit individuelleren Zeitverständnis nach. In der Tat wurden in den letzten Jahrzehnten in vielen Bereichen zeitbezogene Regulierungen liberalisiert: zulässige Ladenöffnungszeiten wurden ausgeweitet, die Arbeitszeitgesetzgebung flexibilisiert oder die Absicherung der Zukunft durch den Wohlfahrtsstaat zunehmend auf private Altersvorsorge und Versicherungen übertragen. Weicheren, dezentralen Koordinationsmechanismen kommt für die Synchronisation einzelner Zeitlichkeiten somit eine größere Bedeutung zu.

Ich möchte die Form der Durchsetzung der vorherrschenden zeitlichen Ordnung, wie sie in Normalzeiten dominiert, daher als „zeitbezogenen Ordoliberalismus“ beschreiben. Analog zur Charakterisierung ordoliberaler Wirtschaftsordnungen bezeichne ich damit eine zeitliche Ordnung von Wirtschaft und Gesellschaft, die durch staatliche Maßnahmen gerahmt wird. Bis auf wenige Ausnahmen, etwa für Gefangene, Schulpflichtige und Sozialhilfeempfänger, wird die konkrete Ausgestaltung der jeweils geltenden zeitlichen Ordnung nicht direkt durch staatliche Akteure bestimmt. Die Durchsetzung der oben beschriebenen Charakteristika des kapitalistischen Zeitregimes wird nicht durch Gesetze festgeschrieben oder durch staatliche Akteure vollstreckt. Gemäß der zugrunde liegenden liberalen Maxime werden die eigentliche Allokation von Zeitbudgets, die Orientierung an Vergangenheit, Gegenwart und Zukunft sowie Rhythmen, Geschwindigkeiten und Reihenfolgen in weiten Teilen des wirtschaftlichen und gesellschaftlichen Lebens den Akteuren überlassen. In freiheitlichen Gesellschaften kann jeder und jede also im Prinzip frei über die eigene Zeit verfügen, sie nach Belieben gewinnbringend veräußern oder verbummeln, für die Zukunft planen oder in den Tag hineinleben.

Die konkrete Koordination individueller Zeitlichkeiten erfolgt indes durch dezentrale Institutionen. Neben tradierten und familialen Normen spielen dabei Organisationen – und in kapitalistischen Gesellschaften allen voran: Unternehmen – eine wichtige Rolle (Lee und Liebenau 1999). Durch formale interne Regeln, Abläufe und Sanktionen, aber ebenso durch informelle Konventionen und Erwartungen strukturieren Unternehmen die Zeitlichkeiten ihrer Arbeitnehmer, Manager, Lieferanten und Kunden. Sie organisieren so die Nutzung, die Wahrnehmung und das Denken über Zeit. Organisationen bedingen und synchronisieren viele zeitbezogene Praktiken direkt, haben dabei jedoch immer auch eine Erziehungsfunktion. Was E. P. Thompson (1967) für die Frühphase der Industrialisierung eindrücklich beschreibt gilt auch heute für viele betriebliche Kontexte: Durch ständiges Rekapitulieren und Einüben werden die Logiken von Zeitvermessung, rationaler Zeitnutzung, Beschleunigung und planvoller Zukunftsorientierung internalisiert. Erst durch diese inkorporierte Selbstkontrolle (Elias 1984) gelingt es, die Imperative des kapitalistischen Zeitregimes auch ohne offensichtliche Sanktionen und über den Geltungsbereich der Organisationen hinaus durchzusetzen.

Darüber hinaus kommt der für kapitalistische Gesellschaften spezifischen Institution des Marktes eine wesentliche Funktion für die Durchsetzung der zeitlichen Ordnung zu. Die „unsichtbare Hand des Marktes“ koordiniert nicht nur Angebot und Nachfrage, sondern auch verschiedene Zeitlichkeiten. Sie bestimmt Geschwindigkeiten, Reihenfolgen, Zeitbudgets, Rhythmen und zeitliche Orientierungen von Akteuren und Organisationen, die im Wettbewerb bestehen wollen. Die marktliche Koordination von Zeitlichkeiten führt jedoch nicht nur zu einem potenziellen Zugewinn an Effizienz und Anpassungsfähigkeit, sondern auch dazu, dass zeitliche Autonomie, das heißt der selbstbestimmte Umgang mit Zeit (Goodin et al. 2008), stark ungleich verteilt ist. *Im Prinzip* können alle frei über die eigene Zeit verfügen. In der Realität hängt es jedoch oft von den verfügbaren Ressourcen, sprich dem Kapital der Akteure ab, inwiefern sie sich den Imperativen des kapitalistischen Regimes beugen müssen oder entziehen können. Mit Geld lassen sich Prozesse beschleunigen oder Zeit zurückkaufen; Geld erleichtert es, sich die Zukunft offenzuhalten; wer über ausreichend Geld verfügt, kann andere Akteure dazu zwingen, ihre Rhythmen anzupassen, oder Zeit überbrücken. So zeigt Sarah Sharma (2014), dass Eliten, wie etwa die „frequent business travelers“, nahezu nach Belieben und Anlass be- und entschleunigen können. Diese Fähigkeit wird jedoch nur durch eine Armada von weniger privilegierten Akteuren ermöglicht, die sich den ständig wechselnden Geschwindigkeiten und Rhythmen anpassen müssen und so selbst kaum über zeitliche Autonomie verfügen. Andere warten zu lassen oder zur Eile zu nötigen, ist eine Form von Herrschaft (Auyero 2010; Schwartz 1974; Serafin 2019), die sich vor allem jene Akteure leisten können, die über ausreichend Kapital verfügen. Ungleichheit, als ein grundlegendes Merkmal kapitalistischer Gesellschaften, wird so auch in der Dimension der Zeit sichtbar, und zwar als *ungleiche zeitliche Autonomie*. Die staatliche, d.h. ordoliberale Rahmung der Zeitverhältnisse zielt allenfalls in Ansätzen darauf, diese ungleichen Freiheitsräume auszugleichen, und überlässt die konkrete Ausgestaltung der zeitlichen Ordnung in weiten Teilen marktorientierten und organisationsinternen Koordinationsprozessen.

## Die Pandemie als Kollision entgegengesetzter zeitlicher Logiken

Als im Frühjahr 2020 apokalyptische Bilder zunächst aus dem chinesischen Wuhan und später aus dem italienischen Bergamo die Menschen in Aufruhr versetzten, sahen sich die Regierungen weltweit gezwungen, zur Eindämmung der Pandemie Maßnahmen zu ergreifen, die das ökonomische und gesellschaftliche Leben maßgeblich beeinträchtigten (Hale et al. 2021). Um die Übertragung des Coronavirus zu unterbinden, sollten „soziale Kontakte“^4^ verringert und auf das Nötigste reduziert werden. Lockdowns, Verbote bestimmter Dienstleistungen und Veranstaltungen, Schließungen von Geschäften, Freizeit- und Bildungseinrichtungen, Kontaktbeschränkungen und Ausgangssperren stellten zuvor undenkbare Eingriffe dar, die den freiheitlichen Charakter moderner Wirtschafts- und Gesellschaftssysteme auf vielen Ebenen erschüttert haben.

In der wissenschaftlichen Auseinandersetzung und entlang öffentlicher Diskurse wurde diese Erschütterung und der Ausnahmezustand, der mit ihr einhergeht, häufig als räumliches Phänomen begriffen. Der Gegensatz von Nähe und Distanz, die Wiederkehr von Grenzen oder die Identifikation von Hotspots und Risikogebieten rückten für viele Menschen ins Blickfeld. In einer raumtheoretisch fundierten Betrachtung beschreiben Hubert Knoblauch und Martina Löw (2020) die Pandemie daher als eine „Refiguration von Räumen“. Zum Schutz der Bevölkerung griffen Regierende massiv in die räumliche Ordnung der Gesellschaft ein und definierten soziale Räume auf neue Art und Weise. Diese Refiguration, so die Autoren, sei geprägt von einem Spannungsverhältnis widersprüchlicher räumlicher Logiken: von Öffnung und Schließung, Territorien und Netzwerken, Hierarchie und Heterarchie.

Der folgende Abschnitt erweitert diesen Befund der Refiguration und zeigt auf, dass die staatlichen Maßnahmen zur Bekämpfung von COVID-19 die gesellschaftliche Ordnung nicht nur in der Dimension des Raumes, sondern ebenso in der Dimension der Zeit herausgefordert und – zumindest temporär – erschüttert haben. Zeitliche Strukturen und Zeitlichkeiten, die das moderne Zusammenleben für gewöhnlich prägen, wurden im Ausnahmezustand der Pandemie infrage gestellt. Auch aus zeitsoziologischer Perspektive zeigt sich ein Spannungsverhältnis, das heißt eine Kollision verschiedener Logiken.^5^ Die staatlichen Maßnahmen der Pandemiebekämpfung etablierten eine zeitliche Logik, die die Prinzipien des kapitalistischen Zeitregimes erschweren, unmöglich machen oder ins Gegenteil verkehren (vgl. Suckert 2021). Anstelle von Beschleunigung zeigten sich neue Imperative von Verlangsamung und Geduld; statt Kommodifizierung und expansiver Verwertung von Zeit erlebten wir im Ausnahmezustand der Pandemie eine Redistribution und partielle Dekommodifizierung von Zeitbudgets; und die Zukunft entzog sich der Aneignung und wurde zu einer Sphäre radikaler Unsicherheit (vgl. auch unten Tabelle 1).

Die beschriebene zeitliche Logik der Pandemiebekämpfung wird dabei nicht zuletzt durch die zeitlichen Spezifika der COVID-19-Pandemie bedingt. Staatliches Handeln, das auf die Eindämmung des Virus zielt, muss sich am exponentiellen Wachstum von Ansteckungen, an sich beschleunigenden oder abebbenden Inzidenzen, Inkubationszeiten oder plötzlich auftretenden Virusvarianten orientieren. Gleichwohl leitete sich die zeitliche Logik der Pandemiebekämpfung nicht einfach kausal aus der Zeitlichkeit des Virus ab oder ist gar mit dieser identisch. Die Bekämpfung der Pandemie und ihre inhärente zeitliche Logik bleiben maßgeblich politische Entscheidungen und damit Gegenstand sozialwissenschaftlicher Analyse. Der Paradoxie der Prävention folgend, wurde die Erschütterung der etablierten zeitlichen Ordnung dann auch weniger durch das Virus selbst und die von ihm verursachten Krankheits- und Todesfälle bewirkt, sondern durch staatliche Interventionen, die darauf zielten, die weitreichende und schnelle Ausbreitung von COVID-19 – und damit Krankheit und Tod – abzuwenden. „Nicht das Virus holte die Flugzeuge vom Himmel und stoppte den Spielbetreib in den Fußball-Ligen, sondern das staatliche politische Handeln“, stellt Rosa (2020, S. 195) zu Recht fest. Die folgenden Abschnitte arbeiten die zeitliche Logik der staatlichen Pandemiebekämpfung heraus und zeigen, in welcher Weise sie mit dem kapitalistischen Zeitregime kollidierte.

### Neue Imperative von Verlangsamung und Geduld

**Figure 1:**
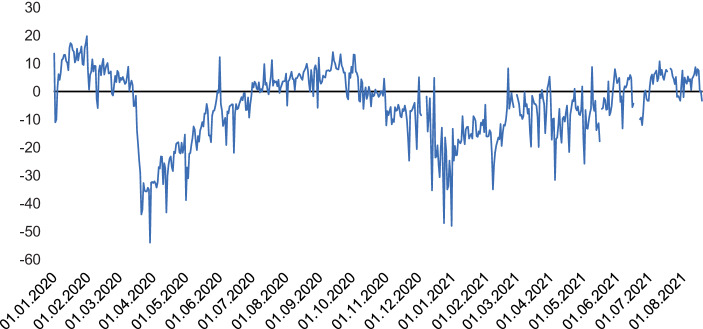

Die sonst gültigen Normen von Wachstum und Beschleunigung schienen mit dem Ausbruch der Krise in den Hintergrund gerückt. Experten wurden nicht müde zu betonen, es ginge im Kampf gegen das Coronavirus darum, zu reduzieren, zu verlangsamen und geduldig zu bleiben. Erstaunlich schnell verhalfen viele Staaten diesen neuen Imperativen von Verlangsamung und Geduld zur Geltung und vollzogen, was immer wieder als „Notbremsung“ bezeichnet wurde: Grenzschließungen, Reisebeschränkungen, Ausgangssperren und die Schließung von Produktions- und Konsumstätten zielten darauf, die Ausbreitung von COVID-19 zu verlangsamen – bremsten dabei jedoch auch das soziale Leben aus. Die Wirkung auf Wirtschaft und Gesellschaft lässt sich an vielerlei Indikatoren nachzeichnen. So ist beispielsweise der internationale Flugverkehr seit März 2020 massiv eingebrochen. Selbst im März 2021 lagen laut der International Air Transport Association sowohl die Anzahl der international bedienten Flugrouten als auch die durchschnittliche Anzahl der monatlichen Flüge je Route noch rund 50 % unter der jeweiligen Marke von März 2019 (IATA 2021). Auch das Verkehrsaufkommen auf deutschen Straßen reduzierte sich während der ersten Welle der Pandemie um fast die Hälfte und blieb selbst 2021 merklich unter dem Niveau von 2019 (BAST 2021). In ähnlicher Weise zeigen Mobilfunkdaten für Deutschland, dass sich die Mobilität in der ersten Welle ab Mitte März 2020 um rund 40 % gegenüber dem Vergleichszeitraum 2019 verringert hat (Abbildung 1); in der zweiten Welle ab Dezember 2020 und der dritten Welle ab März 2021 lag die Mobilität immerhin noch rund 30 beziehungsweise 20 % unter den entsprechenden Werten von 2019 (Statistisches Bundesamt 2021a). Die merkbare Reduktion von Bewegung seit Ausbruch der Pandemie veranschaulicht eine zumindest physische Verlangsamung des gesellschaftlichen Lebens.

Diese reduzierte Dynamik, aber auch die damit einhergehenden Synchronisationsprobleme in Betrieben und Just-in-time-Lieferketten schlugen sich schließlich in einer verringerten Wirtschaftsleistung nieder, einer Größe, die immer auch *pro Zeiteinheit* gemessen wird. Weltweit und speziell für Deutschland brach im Frühjahr 2020 das Bruttoinlandsprodukt je Quartal ein und erholt sich seitdem nur zögerlich. Bezeichnenderweise hat die OECD ihren im Frühjahr 2021 erschienenen Report, der mehr Anstrengungen zur wirtschaftlichen Erholung anmahnt, mit dem Titel „The need for speed“ überschrieben (OECD 2021b).

Auch auf der Mikroebene haben die wiederholten Lockdowns zu einer veränderten Wahrnehmung von Zeit geführt. Zeit wurde von einem abstrakten, aber knappen Gut zu etwas sehr Konkretem, das für manche Menschen auf einmal im Überfluss vorhanden war. Warten wurde zu einer dominanten Alltagspraxis, eine Erfahrung, die für kapitalistisch sozialisierte Menschen neu war. Es musste plötzlich vor Geschäften, Schnellteststationen und an nationalen Grenzen gewartet werden; man hatte sich zu gedulden, bis zur nächsten Lieferung Toilettenpapier, zum ersten verfügbaren Zeitslot im Freibad oder zum ersehnten Impftermin. Dabei mussten verschiedene Akteursgruppen nicht nur unterschiedlich lange warten (siehe unten), sondern auch das Warten an sich wurde sehr unterschiedlich empfunden. Manche bildungsbürgerlichen Milieus mögen die Lockdowns als wohltuende Entschleunigung genossen haben, als Befreiung aus dem rigiden kapitalistischen Zeitregime und einer Kultur des Überflusses. Geduldig zu warten fällt jedoch schwerer, wenn die allgemeine Geschwindigkeitsreduktion auch mit einem kritischen Absinken des eigenen Einkommens einher geht; es erscheint bedrohlicher, wenn die eigene Karriere von empfindlichen Zeitplänen und befristeten Verträgen abhängt. Darüber hinaus wurde auch der schlichte Überfluss an verfügbarer Zeit nicht von allen gleichermaßen als beglückend empfunden. So vervierfachten sich während der ersten Welle in Deutschland beispielsweise Internetsuchanfragen, die den Ausdruck „Langeweile“ enthielten, und blieben während der nachfolgenden Wellen auf einem hohen Niveau (vgl. Google Trends). Wenngleich die Aussagekraft solcher Suchanfragen mit Vorsicht zu genießen ist,^6^ deuten sie doch darauf hin, dass Erfahrungen von Langeweile (Brissett und Snow 1993; Ohlmeier et al. 2020) im Ausnahmezustand der Pandemie zugenommen haben. Die Verlangsamung des wirtschaftlichen und gesellschaftlichen Lebens und die Abkehr von Beschleunigungsimperativen scheinen auch als Entleerung und Entwertung der eigenen Lebenszeit empfunden worden zu sein.

### Dekommodifizierung und Umverteilung von Zeitbudgets

**Figure 2:**
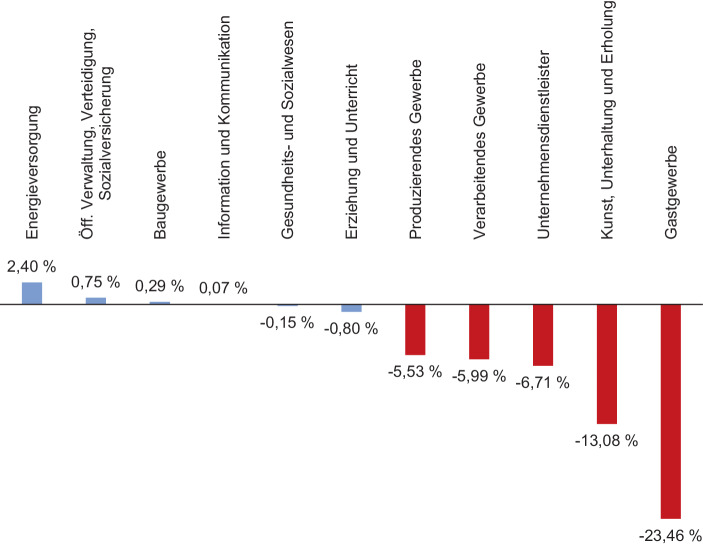

Die staatlichen Anstrengungen zur Eindämmung der Pandemie haben zudem eine Umverteilung von Zeit erzwungen und damit Tendenzen der Kommodifizierung und expansiven Verwertung von Zeit, die dem kapitalistischen Zeitregime in Normalzeiten inhärent sind, abgeschwächt. Den beiden für die kapitalistische Akkumulation unerlässlichen Prozessen von Produktion und Konsum wurde im Zuge der Pandemie ein erheblicher Anteil der verfügbaren Zeit entzogen. So schätzt die Internationale Arbeitsorganisation (ILO 2021), dass weltweit 2020 rund 8,8 % weniger Arbeitsstunden erbracht wurden als noch im Jahr zuvor – was einem Äquivalent von 225 Millionen verlorenen Vollzeitstellen entspricht. Eine erhebliche Reduktion von Arbeitszeit lässt sich auch in Deutschland erkennen, wo die ILO von rund 6,3 % weniger erbrachten Arbeitsstunden beziehungsweise dem Äquivalent von 2,4 Millionen verlorenen Vollzeitstellen ausgeht. Jedoch unterscheidet sich diese Dekommodifizierung von Arbeitszeit markant nach Sektoren. Abbildung 2 zeigt die errechneten Arbeitnehmerstunden für ausgewählte Wirtschaftssektoren, für die bereits Daten für 2020 vorliegen (Statistisches Bundesamt 2021e). Obwohl es sich hierbei um stark aggregierte Daten handelt, die jeweils sehr verschiedene wirtschaftliche Unterkategorien zusammenfassen, lassen sich klare Unterschiede erkennen: Die entlohnten Arbeitsstunden in den Bereichen Produzierendes Gewerbe, Verarbeitendes Gewerbe, Unternehmensdienstleister, aber auch ganz besonders in den Bereichen Kunst, Unterhaltung und Erholung sowie Gastgewerbe haben pandemiebedingt abgenommen (rot bzw. schwarz dargestellt). Dies lässt sich nicht zuletzt auf politisch forcierte Schließungen von Betriebsstätten zurückführen. Hingegen haben andere Bereiche kaum Einbußen oder sogar einen leichten Zuwachs hinsichtlich der Arbeitnehmerstunden zu verzeichnen (blau bzw. grau dargestellt): Beispielsweise wurde das Gesundheitssystem stärker belastet, dem Bereich Information und Kommunikation kam durch den pandemiebedingten Digitalisierungsschub größere Bedeutung zu, das Baugewerbe war weniger durch staatlich verordnete Betriebsschließungen betroffen und erfuhr zudem eine höhere Nachfrage, öffentliche Verwaltung und Energieversorgung mussten schließlich die gerade im Ausnahmezustand dringend nötige öffentliche Infrastruktur aufrechterhalten. Für zahlreiche Unterkategorien, die diesen breiten Kategorien zugerechnet werden, ist daher tatsächlich von einer Ausweitung der Arbeitszeit auszugehen. Wenngleich sich auf der Makroebene somit insgesamt eine Dekommodifizierung von Arbeitszeit konstatieren lässt, geht diese doch mit einer zeitlichen Redistribution zwischen Sektoren einher, die in einzelnen Wirtschaftsbereichen sowohl zu einer Intensivierung als auch zu einer (relativen) Expansion der Arbeitszeit führt.

Eine Umverteilung von Zeitbudgets lässt sich im Zuge der Coronakrise jedoch auch für einzelne Lebensbereiche erkennen. Die wiederholten Lockdowns haben vielen Menschen verdeutlicht, wie stark ihre Lebensführung durch Erwerbsarbeit und Konsum geprägt ist – wird Beides durch staatliche Eingriffe erschwert oder entfällt es ganz, entstehen beachtliche zeitliche Freiräume. Gefüllt wurden diese einerseits durch digital vermittelten Konsum, wie die beeindruckenden Wachstumsraten der digitalen Unterhaltungsbranche belegen: So verzeichnete etwa der deutsche Markt für Computer- und Videospiele 2020 dem Branchenverband Game (Game 2021) zufolge ein Wachstum von 36 %, der Video-on-Demand-Anbieter Netflix ein Umsatzwachstum von 24 %. Ebenso erlebten die Anbieter von Sport-Apps enorme Zuwächse (SensorTower 2021). Andererseits entdeckten viele Menschen weniger kommerzialisierte Formen des Zeitvertreibs: Spazierengehen, Musizieren, Nähen, Brot backen, Heimwerken und Gartenarbeit wurden zu weitverbreiteten Trends. Internetsuchanfragen mit dem String „selber machen“ verdoppelten sich mit dem ersten Lockdown und blieben in den nachfolgenden Wellen so prominent, wie sonst nur in der Adventszeit (Google Trends). Zeit, die vormals für Konsum oder Erwerbsarbeit genutzt wurde, wurde so der kapitalistischen Verwertung entzogen.

Darüber hinaus zeigt sich jedoch auch eine Umverteilung von Zeitbudgets hin zu unbezahlter, dekommodifizierter Sorgearbeit. Die Schließung von Bildungs- und Betreuungseinrichtungen zählte weltweit zu einer der ersten Maßnahmen, um der Pandemie Herr zu werden. Laut dem „COVID-19 Government Response Tracker“ der Universität Oxford (Hale et al. 2021, 2020) war im Frühjahr 2020 weltweit nur in einer Handvoll Ländern der Schulbesuch gestattet, und auch im weiteren Verlauf der Pandemie gehörten Schul- und Kitaschließungen in nahezu allen Ländern zum gängigen Instrumentarium der Pandemiebekämpfung. Auch in Deutschland mussten Kinder zuhause lernen, wurden Ferien verlängert oder lediglich Wechselunterricht gestattet. Laut OECD (2021a) waren deutsche Kitas seit dem Frühjahr 2020 an 61 von 270 betrachteten Tagen geschlossen; Schüler*innen konnten lediglich an 90 von 270 Tagen die Schule regulär besuchen. Ähnlich gestaltete sich die Situation in Werk- und Förderstätten für Menschen mit Behinderung sowie in Einrichtungen der Tagespflege für ältere Menschen. Auch waren, zumindest im ersten Lockdown, viele haushaltsnahe Dienstleistungen nicht möglich. Durch die staatlich verordneten Schließungen brach so eine wesentliche Infrastruktur weg, die es Familien gewöhnlich erlaubt, die zeitintensive Sorgearbeit auszulagern und so Zeit zurückzugewinnen, um sie der kapitalistischen Verwertung als Erwerbsarbeit zuzuführen. Durch den Wegfall dieser Infrastruktur wurde Zeit in vielen Familien noch knapper als ohnehin schon. Eltern – und noch häufiger Mütter (Zucco und Lott 2021) – sahen sich gezwungen, ihre Arbeitszeit zu reduzieren, um der nötigen Sorgearbeit nachzukommen. Die staatlich erzwungene Dekommodifizierung und Redistribution von Zeitbudgets legte so einerseits offen, wie sehr der zeitliche Expansionismus, das heißt die Tendenz, möglichst viel Zeit kapitalistisch verwertbar zu machen, in Normalzeiten andere Lebensbereiche an den Rand drängt und ihnen die verfügbare Zeit entzogen hat. Andererseits hat das Wegbrechen dieser Infrastruktur, die es niedrigschwellig erlaubt, sich im Alltag „freizukaufen“, um selbst von der beruflichen Kommodifizierung von Zeit zu profitieren, die ungleiche Verteilung zeitlicher Autonomie – zum Beispiel zwischen Geschlechtern und sozialen Milieus – zusätzlich verschärft.

### Von der gestaltbaren Zukunft zur radikalen Unsicherheit

Ebenso wurde die dem kapitalistischen Zeitregime inhärente Vorstellung einer kalkulierbaren Zukunft im Zuge der Pandemie infrage gestellt. Da sie die einfache Fortschreibung des Status quo undenkbar macht, irritiert jede Krise etablierte Zukunftserwartungen zunächst grundlegend (Ergen und Suckert 2021). Die Coronakrise ist jedoch durch Unisicherheiten gekennzeichnet, die in Ausmaß und Fortdauer bemerkenswert erscheinen. Die Pandemie beeinträchtigt nahezu alle Länder und Lebensbereiche zugleich, Entwicklungen und Interferenzen lassen sich kaum absehen. Virusvarianten, neue oder revidierte medizinische Erkenntnisse oder Simulationsmodelle tragen zu dieser Verunsicherung bei – sie veranlassen vor allem aber auch politische Entscheider, getroffene Maßnahmen der Pandemiebekämpfung immer wieder zu überdenken, anzupassen oder auszuweiten. So sind das Virus und seine gesundheitlichen Gefahren zwar ursächlich für die Krise, ein Großteil der pandemiebezogenen Unwägbarkeiten wird jedoch nicht zuletzt durch die wechselnden staatlichen Maßnahmen zur Abwehr dieser Gefahren hervorgerufen. Unter welchen Bedingungen wird der Konzertbesuch, die Familienfeier oder das Arbeitstreffen im nächsten Monat noch erlaubt sein? Welche Hygienevorschriften werden Betriebe erfüllen müssen? Wird es in zwei Wochen noch gestattet sein zu reisen, die Kinder in den Kindergarten zu bringen oder nach 21 Uhr das Haus zu verlassen? Angesichts des Eindrucks stetig wechselnder staatlicher Rahmenbedingungen wurde die Zukunft im Zuge der Pandemie von einem Raum der Möglichkeiten zu einer Sphäre radikaler Unsicherheit, in der sich allenfalls noch auf Sicht navigieren lässt.

Das Ausmaß der Unsicherheit zeigt sich auch daran, dass Instrumente, die im Kapitalismus gewöhnlich dafür genutzt werden, die Zukunft zu „domestizieren“, an ihre Grenzen stießen. Dies gilt für Geschäftspläne ebenso wie für Versicherungen – die bei pandemiebedingten Ausfällen meist nicht einsprangen, denn, so der Branchenverband GDV (2020), „die Folgen einer Pandemie sind schwer zu kalkulieren“ und damit nicht als Risiko fass- und versicherbar. Auch makroökonomischen Vorhersagen, die in Normalzeiten einen wichtigen Orientierungspunkt für Wirtschaft und Politik darstellen, fiel es schwer, die Zukunft greifbar zu machen. Zahlreiche Prognoseinstitute mussten im Frühjahr 2020 ihre Vorhersagen in kurzen Abständen korrigieren oder Veröffentlichungen verschieben. Da sich die Zukunft der Krise nicht mehr als Verlängerung der Vergangenheit kalkulieren ließ, sahen sich Konjunkturforscher gezwungen, alternative Indikatoren und Methoden zu entwickeln, um zumindest die Gegenwart erfassen zu können (Foroni et al. 2020).

**Figure 3:**
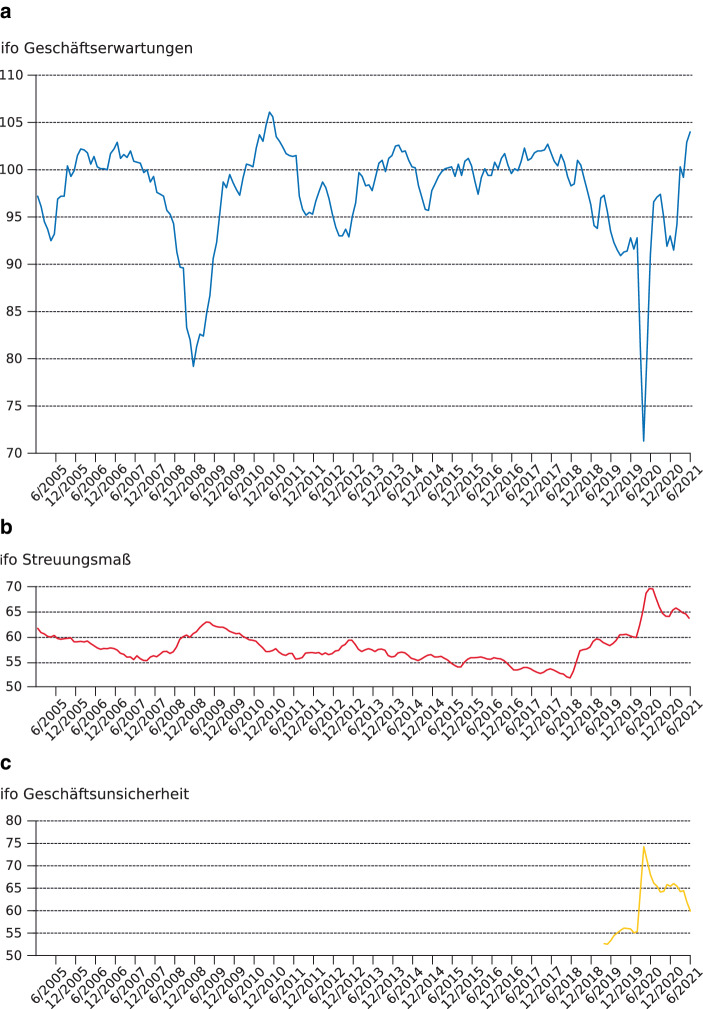

Die Schwierigkeit, die Zukunft zu kalkulieren, zeigt sich aber auch auf der Ebene der privaten Haushalte und Unternehmen, die oft kaum in der Lage waren, die nächsten Monate, Wochen oder auch nur Tage zu planen. Die laufenden Umfragen des ifo Instituts zum Geschäftsklima in Deutschland zeigen einerseits, dass sich die Geschäftserwartungen von Unternehmen mit Ausbruch der Coronakrise zunächst stark eingetrübt, dann aber wieder erholt haben (vgl. Abbildung 3a). Darüber hinaus wurden Unternehmer*innen ab April 2019 danach gefragt, wie schwer es ihnen fällt, Geschäftserwartungen zu äußern (vgl. Lautenbacher et al. 2020). Der daraus abgeleitete Index macht deutlich, dass Unternehmen über die Krise hinweg weit weniger in der Lage waren, überhaupt eine Einschätzung zu ihren Zukunftsaussichten abzugeben (Abbildung 3c). Der Blick auf das Streuungsmaß für die Geschäftserwartungen zeigt, dass die Erwartungen der Befragten stark voneinander abweichen (Abbildung 3b; vgl. auch Grimme 2020). In der Krise gelang es somit nicht mehr wie gewöhnlich, ein gemeinsames Verständnis der Zukunft zu etablieren. Die Zukunft wurde auch in den Unternehmen zu einer kaum mehr handhabbaren Größe. Immer wieder wurden daher öffentliche Forderungen nach verlässlichen staatlichen Regelungen laut, nach politischer Orientierung und klaren Zukunftsperspektiven. „Diese Unsicherheit“, kommentierte etwa der DIW-Präsident Marcel Fratzscher (2021), „ist Gift für die Wirtschaft“.

Die Wahrnehmung der Zukunft als einer Sphäre radikaler Unsicherheit erscheint für kapitalistische Gesellschaften vor allem deshalb problematisch, weil die Vorstellung einer gestaltbaren Zukunft Voraussetzung für deren Aneignung in der Gegenwart ist. Eine erwartete unsichere Zukunft hat reale Konsequenzen in der Gegenwart. Die verringerte Bereitschaft von Unternehmen, in ihre Ausrüstung zu investieren (2020 −10 %; Statistisches Bundesamt 2021c), sowie der merkliche Rückgang von Unternehmensgründungen im Jahr 2020 können in diesem Sinne interpretiert werden: Unternehmen haben darauf verzichtet, die Zukunft aktiv zu gestalten. Allerdings zeigen sich auch hier Unterschiede: Während die Gründungen von größeren Unternehmen um 4,5 % zurückgingen, verringerte sich die Zahl der neu gegründeten Kleinunternehmen innerhalb eines Jahres um stattliche 17,3 % (Statistisches Bundesamt 2021b). In der Krise wird die Zukunft zwar für alle zu einer Sphäre größerer Unsicherheit – wie bedrohlich diese Unsicherheit wirkt und in welchem Maße sich Akteure noch zutrauen, sich diese unsichere Zukunft anzueignen, hängt jedoch wesentlich von den verfügbaren Ressourcen ab.

In der Zusammenschau der letzten drei Abschnitte zeigt sich, dass die restriktiven staatlichen Maßnahmen zur Pandemiebekämpfung einer zeitlichen Logik folgen, die dem kapitalistischen Zeitregime in weiten Teilen zuwiderläuft und dessen grundlegende Prinzipien infrage stellt. Viele der sozialen und ökonomischen Verwerfungen dieses Ausnahmezustandes lassen sich daher auch als Folgen der Kollision dieser unterschiedlichen zeitlichen Logiken verstehen. Diese Kollision hat nicht zuletzt Bruchstellen und Paradoxien des kapitalistischen Zeitregimes aufgezeigt.

Allerdings erscheint es mir voreilig zu schließen, der Staat habe diese zeitliche Logik *gegen* den Kapitalismus durchgesetzt, wie dies Rosa suggeriert (2020, S. 201 f.). Die Eindämmung der Pandemie – und die damit einhergehende zeitliche Logik – wurde auch und gerade von Vertretern des Kapitals vehement eingefordert. Wirtschaftsfreundliche Initiativen wie No-COVID (Baumann et al. 2021) warnten vor vorschnellen Lockerungen und argumentierten damit, dass gerade eine rigorose Pandemiebekämpfung im Interesse der Wirtschaft sei. Eine grassierende Pandemie, so das Kalkül, wäre weitaus kostspieliger und würde das Wirtschaftswachstum nachhaltiger schädigen. Dabei wurde häufig auch auf Länder wie Brasilien verwiesen, die es ablehnten, die Wirtschaft auszubremsen, und in der Folge nicht nur die Überlastung der Gesundheitssysteme, Krankheit und Tod, sondern auch erhebliche ökonomische Verwerfungen erlebten. Die „Notbremse“ wurde hierzulande als unausweichlich erachtet, aber gerade auch, um kapitalistisches Wachstum langfristig zu sichern. Die staatlichen Maßnahmen zur Pandemiebekämpfung haben das kapitalistische Zeitregime somit in der Tat herausgefordert, indem sie die Kommodifizierung und expansive Verwertung von Zeit, die Beschleunigungsimperative und die Aneignung der Zukunft erschwert, unmöglich gemacht oder ins Gegenteil verkehrt haben – all dies geschah jedoch in der Erwartung einer möglichst baldigen Rückkehr zu normalen Zeiten. Die Neuordnung der Zeit war selbst nur auf Zeit angelegt.

Die Hoffnung, dass die staatliche Pandemiepolitik einer längerfristigen Einhegung des kapitalistischen Zeitregimes dient oder sich ein „Spalt[] zwischen staatlichem Handeln und Kapitalakkumulation“ (Rosa 2020, S. 202) abzeichnet, erscheint somit bereits fragwürdig, wenn man nur jene politischen Maßnahmen in den Blick nimmt, die auf die reine Pandemiebekämpfung, das heißt auf die Verhinderung von Ansteckungen ausgerichtet sind. Sie wird noch zweifelhafter, wenn man den zweiten Typus pandemiepolitischer Interventionen betrachtet, der darauf zielt, die Folgeschäden der Pandemie für Wirtschaft und Gesellschaft abzufedern. Wie ich im nächsten Abschnitt zeigen werde, lassen sich viele dieser staatlichen Maßnahmen aus einer zeitsoziologischen Perspektive auch als Strategien verstehen, zwischen den zeitlichen Logiken von Pandemiebekämpfung und Kapitalismus zu vermitteln und den Kapitalismus somit, trotz Pandemiebekämpfung, „im Spiel“ zu halten.

## Von der Rückkehr des Staates zur Neuordnung der Zeit?

Die beschriebene zeitliche Logik der Pandemiebekämpfung, die sich nicht zuletzt auch an den zeitlichen Spezifika des Coronavirus orientiert, wurde unmittelbar durch staatliche Maßnahmen durchgesetzt. Im Zuge der Pandemie lässt sich somit eine gewisse Rückkehr des Staates als zeitpolitische Ordnungsmacht erkennen. Die Festlegung von Rhythmen, Geschwindigkeiten, zeitlichen Orientierungen und Reihenfolgen wurde vielerorts nicht länger marktorientierten Koordinationsprozessen oder der organisationalen Selbstverwaltung überlassen, sondern in gesteigertem Maße auf politische Entscheidungsprozesse verlagert.

Allerdings griff der Staat auch jenseits der reinen Pandemiebekämpfung aktiver als zuvor in die zeitliche Ordnung von Wirtschaft und Gesellschaft ein. Aus zeitsoziologischer Perspektive lassen sich viele der staatlichen Maßnahmen, die über die reine Eindämmung des Infektionsgeschehens hinaus gehen, als Versuch verstehen, die Kollision zwischen den zeitlichen Imperativen der Pandemiebekämpfung und dem kapitalistischem Zeitregime abzufedern. Politische Interventionen, die darauf zielen, die ökonomischen und gesellschaftlichen Verwerfungen der Pandemie zu mildern, sind im Kern auch Strategien^7^, um zwischen den gegensätzlichen zeitlichen Logiken zu vermitteln (vgl. Tabelle 1). Sie erlauben es, den zeitlichen Erfordernissen der Pandemiebekämpfung gerecht zu werden, ohne dem kapitalistischen Zeitregime langfristig zu entsagen. Hinsichtlich der verschiedenen Strategien, die ich im Folgenden darstellen möchte, sticht jedoch ins Auge, dass die Kollision der zeitlichen Logiken nicht für alle Akteursgruppen in der selben Art und Weise abgefedert wird. Zeitbezogene Ungleichheiten schreiben sich so trotz und wegen der Rückkehr des Staates als zeitpolitische Ordnungsmacht fort.

**Table 1:** 

Charakteristika des kapitalistischen Zeitregimes		Zeitliche Logik der Pandemiebekämpfung	Strategien der Vermittlung
Beschleunigung	vs.	Neue Imperative von Verlangsamung und Geduld	Staatlich definierte Reihenfolgen und Geschwindigkeiten Anhalten der Zeit
Kommodifizierung und expansive Verwertung von Zeit	vs.	Dekommodifizierung und Umverteilung von Zeitbudgets	Staatliche Erleichterung von Dekommodifizierung
Aneignung der Zukunft	vs.	Zukunft als radikale Unsicherheit	Überbrückung von Zeit und Wiederherstellung von Planbarkeit Staatliche Definition von Zukunftsfähigkeit

Erstens unterlagen im Zuge der Pandemie viele *Geschwindigkeiten und Reihenfolgen* nicht mehr der Logik von Zahlungsbereitschaft und -fähigkeit oder Angebot und Nachfrage, sondern wurden von der Politik festgelegt. Staatliche Akteure bestimmten nicht nur einen umfassenden Stillstand, sondern verfügten auch, inwiefern Wirtschaft und Gesellschaft wieder Fahrt aufnehmen durften. Welche Bereiche wann, unter welchen Bedingungen und mit welcher Auslastung öffnen durften, wer vorrangig Zugang zu Tests bekommen sollte, wer zuerst geimpft werden konnte oder länger auf den befreienden Schutz warten musste – all dies unterlag nicht marktlicher Koordination und unternehmerischer Planung, sondern staatlicher Direktion. Staatliche Eingriffe und Subventionen entschieden darüber, welche Bereiche noch im Lockdown ausharren mussten, allmählich zur kapitalistischen Beschleunigungslogik zurückkehren konnten oder gar in den staatlich beförderten Modus des „Aufholens“ umschalten durften. Die bereits aus der Finanzkrise bekannte, aber nun neu interpretierte Kategorie der „Systemrelevanz“ spielte für die Legitimation dieser Reihenfolgen eine wichtige Rolle. Dabei wurden zumindest zeitweilig auch Wirtschaftsbereiche und Berufsgruppen als systemrelevant aufgewertet, denen sonst wenig wirtschaftspolitische Beachtung zuteilwird. Gleichwohl zeichnete sich ab, dass die politisch bestimmten Geschwindigkeiten und Reihenfolgen maßgeblich auch widerspiegeln, in welchem Maß Akteursgruppen in der Lage sind, Einfluss auf politische Entscheidungen zu nehmen, und über Interessenvertretungen mit entsprechenden Ressourcen und Kontakten verfügen. Dass beispielsweise die Vereine der Fußballbundesliga sehr früh den Spielbetrieb wieder aufnehmen durften und bevorzugt Tests erhielten, während Sportstätten für Kinder noch lange geschlossen bleiben mussten, lässt sich nur schwer mit dem Argument der Systemrelevanz, wohl aber mit erfolgreicher politischer Lobbyarbeit erklären.

Zweitens zielen staatliche Maßnahmen darauf, die pandemiebedingt erforderliche *Dekommodifizierung von Zeit zu erleichtern* und eine größere zeitliche Autonomie zu ermöglichen. Eine flexiblere Aufteilung von Lohnarbeits- und Sorgearbeitszeiten wurde dadurch möglich, dass Arbeitgeber verpflichtet wurden, ihren Mitarbeitern Heimarbeit anzubieten. Die Erhöhung der möglichen Kinderkrankentage und deren Ausweitung auf Zeiten ausgefallener Kinderbetreuung erlaubt es Eltern, ihre Arbeitszeit zu reduzieren, um sich stattdessen der Sorgearbeit zu widmen. Das entsprechende Kinderkrankengeld und in geringerem Umfang die Elternentschädigung nach Infektionsschutzgesetz sowie pandemiebezogene Kinderboni kompensieren zumindest teilweise die finanziellen Einbußen durch die Dekommodifizierung der Lohnarbeitszeit (BMFSFJ 2021a). In ähnlicher Weise zielt auch das Kurzarbeitergeld, welches im Zuge der Pandemie in noch nie da gewesenem Ausmaß in Anspruch genommen wurde, darauf, eine unfreiwillige Reduktion von Lohnarbeitszeit finanziell abzufedern. Die Möglichkeit zur Arbeitszeitreduktion ist jedoch gleichzeitig als Versuch zu verstehen, einer noch umfassenderen Dekommodifizierung – im Sinne einer Zunahme der Arbeitslosigkeit – entgegenzuwirken. Wenngleich derartige politische Eingriffe den Druck reduzieren, Zeit gewinnbringend zu kommodifizieren, und somit Freiräume für andere Lebensbereiche schaffen, kommen sie keinesfalls allen Bürger*innen in gleichem Maße zugute – und verstärken so teilweise bestehende Ungleichheiten. So sind etwa zusätzliche Kinderkrankentage nur für gesetzlich Krankenversicherte vorgesehen, und Beschäftigte mit einem Minijob haben keinen Anspruch darauf (Minijob-Zentrale 2021). Zudem haben Frauen in größerem Ausmaß als Männer ihre Arbeitszeit reduziert und somit Lebenszeit dekommodifiziert – was bestehende Geschlechterunterschiede hinsichtlich Einkommen und Rentenansprüche weiter verschärft (Bonin et al. 2021; Zucco und Lott 2021).^8^

Das bereits angesprochene Instrument der Kurzarbeit erlaubt es Arbeitnehmern, die Dekommodifizierung von Arbeitszeit finanziell zu kompensieren. Es zielt jedoch, drittens, auch darauf, *Zeiten der Unsicherheit zu überbrücken* und die* Planbarkeit der Zukunft *auch angesichts unabsehbarer staatlicher Restriktionen zu gewährleisten. Der Rückgriff auf staatlich subventionierte Kurzarbeit erleichtert es Unternehmen, auszuharren und zu warten. Er ermöglicht ihnen, den Status quo ihrer Beschäftigungsstruktur solange zu erhalten, bis sich die Zukunft wieder besser kalkulieren lässt und eine Einschätzung der langfristigen Auswirkungen sinnvoll möglich ist. Dass im Zuge der Pandemie das Instrument der Kurzarbeit auch für Zeitarbeitsfirmen geöffnet wurde, das heißt für jene Branche, die ihr Geld gewöhnlich damit verdient, Arbeitnehmer flexibel zu verleihen und so die Unwägbarkeiten der Zukunft für andere Betriebe abzufedern, verdeutlicht einmal mehr das enorme Ausmaß der pandemiebedingten Unsicherheit. In diesem Sinne zielt eine Vielzahl wirtschaftspolitischer Maßnahmen (BMWi und BMF 2021) darauf, unsichere Zeiten zu überbrücken. Franz-Xaver Kaufmann beschrieb die Herstellung von Sicherheit als „Vernichtung der Zeitlichkeit der Zukunft“ (1973, S. 157). Absicherung bedeutet die Genese einer Welt, in der sich das, was sein wird, unmittelbar aus dem ableiten lässt, was ist. Die Unwägbarkeit der Zukunft soll schon im Hier und Jetzt auf- und abgefangen werden. Die zahlreichen wirtschaftspolitischen Programme der Bundesregierung, die schon in ihrem Namen erkennen lassen, dass sie als „Überbrückungshilfen“ gemeint sind oder der „Stabilisierung“ dienen, zielen darauf, die besonders in der Krise offensichtlich werdende Zeitlichkeit der Zukunft für Unternehmen zu reduzieren. Durch Bürgschaften, garantierte Kredite, Zuschüsse und Rekapitalisierungen im Umfang von (bislang) 129 Milliarden Euro (BMWi 2021) versucht der Staat, zumindest kurz- und mittelfristig Planbarkeit zu ermöglichen. Dabei geht es unmittelbar zwar immer auch darum, Liquidität zu erhalten und finanzielle Ausfälle und Verwerfungen auszugleichen, die durch Maßnahmen zur Pandemiebekämpfung entstanden sind. Vor allem dienen diese Maßnahmen aber auch dazu, Zuversicht zu vermitteln, das heißt die Vorstellung zu nähren, dass die Zeit der Unsicherheit überstanden werden kann und eine neuerliche Aneignung der Zukunft möglich sein wird. Die gleiche Logik wohnt auch staatlichen Nothilfen für Familien oder dem vereinfachten Zugang zur Grundsicherung inne. Staatliches Handeln zielt in der Krise wesentlich darauf ab, zumindest ein Mindestmaß des für den Kapitalismus so wichtigen Vertrauens in die Zukunft aufrechtzuerhalten.

Indem der Staat versucht, die Zukunft wenigstens partiell abzusichern, werden jedoch, viertens, *Fragen der Zukunftsfähigkeit zu politischen Entscheidungen* umgedeutet. In normalen Zeiten befinden in vielen Bereichen marktliche Koordinationsprozesse über das Zukunftspotenzial von Unternehmen, Akteursgruppen oder Innovationen. In der Pandemie wurde deren Zukunftsfähigkeit – und damit nicht zuletzt deren Zugang zu Ressourcen in der Gegenwart – Gegenstand politischer Abwägungen. Bürgschaften und erleichterte Kredite stellen ein wesentliches wirtschaftspolitisches Instrument der Bundesregierung zur Abfederung pandemiebedingter ökonomischer Verwerfungen dar. Der Staat bürgt hier wortwörtlich für die Zukunftsfähigkeit der Unternehmen. Er ermöglicht ihnen genau dadurch die Chance auszuharren, bis die Zukunft wieder Fahrt aufnimmt. Ebenso lässt sich der finanzielle und administrative „Booster“, den einzelne Pharmazieunternehmen durch staatliche Förderung erhalten haben, als Wette auf das Zukunftspotenzial dieser Firmen verstehen. Den politischen Entscheidern erscheint jedoch nicht jede Zukunft in gleicher Weise förderungswürdig. Branchen, die traditionell eine enge Beziehung zum politischen Betrieb haben, wurden stärker begünstigt. Während beispielsweise die Zukunft der Lufthansa schon zu Beginn der Krise mit rund neun Milliarden Euro durch den Staat gestützt wurde, wurden die Zukunftsängste von Soloselbstständigen und Kulturschaffenden in den ersten Runden der Überbrückungshilfen nicht adäquat berücksichtigt. Ebenso lässt sich das mit zwei Milliarden Euro veranschlagte Aktionsprogramm, das etwa 14 Millionen Kindern und Jugendlichen ermöglichen soll, pandemiebedingte Entwicklungs- und Lerndefizite für die Zukunft nach Corona „aufzuholen“ (BMFSFJ 2021b), im Vergleich zur massiven Wirtschaftsförderung allenfalls noch als symbolischer Akt verstehen. In den politischen Entscheidungen, wessen Zukunft erhalten oder gar beschleunigt werden muss, wird somit nicht zuletzt eine Hierarchisierung von Wirtschafts- und Gesellschaftsbereichen deutlich.

Schließlich versucht, fünftens, ein weiteres Set an staatlichen Maßnahmen, gewissermaßen durch das vorübergehende *Anhalten der Zeit* die Kollision der zeitlichen Logiken von Pandemie und Kapitalismus abzufedern. Veränderte gesetzliche Regelungen verlängerten Fristen, pausierten die Fälligkeiten von Forderungen und unterbrachen so den gewöhnlichen Lauf der Zeit. Die Zeit der Krise wurde damit als Zeit deklariert, die nicht in gewöhnlicher Weise zählt. In vielen Bereichen wurden die Mühlen der Bürokratie angehalten und scheinbar unabdingbare Verwaltungsprozesse ausgesetzt. So wurde es beispielsweise ermöglicht, Steuerzahlungen zu „stunden“, das heißt deren Fälligkeit in die Zukunft zu verschieben, oder sie als Ratenzahlungen auf eine noch weiter entfernte Zukunft zu verteilen (BMF 2021). Zudem wurde die Vollstreckung von Steuernachzahlungen zeitweise ausgesetzt, und in zahlreichen Verwaltungsprozessen wurde ein Zahlungsverzug nicht mehr mit Säumniszinsen bestraft. Diese Aufhebung von Zinsen, also des monetären Preises der Zeit, machte die Krise gewissermaßen zu einer Zeit, die verstreichen kann, ohne dass sie etwas kostet. In ähnlicher Weise sorgte die Aussetzung der Insolvenzantragspflicht dafür, dass die kapitalistische Dynamik der kreativen Zerstörung „eingefroren“ wurde. Tatsächlich registrierte das Statistische Bundesamt für das Jahr 2020 rund 30.000 Insolvenzen weniger als noch im Vorjahr, das ohnehin schon von einem sehr niedrigen Insolvenzniveau geprägt war (Statistisches Bundesamt 2021d). Die sichtbaren ökonomischen Schäden der Pandemie wurden so in die Zukunft vertagt. Die Aussetzung der Antragspflicht verhindert zwar nur formell-rechtlich die Insolvenz, verschafft den betroffenen Unternehmen jedoch Zeit, in der die gewöhnlichen Dynamiken außer Kraft sind. Die Zeit wurde jedoch nicht nur in staatlichen Verwaltungsprozessen angehalten, sondern auch im Hinblick auf einige privatrechtliche Forderungen. So wurden im Frühjahr 2020 COVID-19-bedingte Mietzahlungsrückstände vorübergehend nicht mehr als Grund anerkannt, Mieter*innen zu kündigen (BMJV 2021). Die Pflicht zur Zahlung der Miete wurde dadurch zwar nicht aufgehoben, ein Verzug bei der Zahlung blieb aber zunächst ohne Folgen. Auch hier zählte die Zeit nicht mehr in gleicher Weise, und Mieter*innen wurde stattdessen Zeit gegeben, die Mietzahlung in die Zukunft zu verschieben. Ähnlich ermöglichten befristete Regulierungen es Privatpersonen, ausstehende Darlehensraten oder Forderungen für die Grundversorgung mit Wasser, Strom oder Telefon für einige Monate aufzuschieben. Das staatlich beförderte Anhalten der Zeit in klar definierten Bereichen erlaubte es Akteuren, sich für kurze Zeit von den gewöhnlichen kapitalistischen Dynamiken zu entkoppeln, um die pandemiebedingten Verwerfungen wenigstens zum Teil abzufangen.

In der Zusammenschau zeigt sich, dass viele der staatlichen Maßnahmen zur Abmilderung sozialer und ökonomischer Härten auch als zeitbezogene Praktiken verstanden werden können. Sie vermitteln zwischen den verschiedenen zeitlichen Logiken der Pandemiebekämpfung und des Kapitalismus. Der Staat ist somit nicht nur in der Lage, für sich selbst, wie Wolfgang Streeck (2013) reklamiert, „Zeit zu kaufen“, sondern er verschafft in der Krise auch seinen Bürger*innen Zeit, indem er als zeitpolitische Ordnungsmacht auftritt. Die Erschütterung der zeitlichen Ordnung wird dadurch – wenn auch nicht für alle Akteure in gleicher Weise – abgefedert. Im Zuge dieses Abfederns werden neue zeitliche Praktiken und Möglichkeiten der staatlichen Regulierung erprobt. Der daraus resultierenden Neuordnung der Zeit ist aber immer schon der Status eines Ausnahmezustands und damit implizit die Rückkehr zum kapitalistischen Zeitregime eingeschrieben. Der „zurückgekehrte Staat“ ist, wie Klaus Dörre (2020) treffend bemerkt, lediglich ein „Ausnahmestaat“.

## Diskussion und Ausblick

Die zeit- und wirtschaftssoziologische Betrachtung der Pandemie hat deutlich gemacht, dass COVID-19 die zeitliche Ordnung der Gesellschaft erschüttert hat. Einerseits widersetzt sich die zeitliche Logik der Pandemiebekämpfung dem sonst vorherrschenden kapitalistischen Zeitregime: Kapitalistische Prinzipien von Kommodifizierung und expansiver Verwertung von Zeit, Beschleunigung sowie Aneignung der Zukunft kollidierten mit der Dekommodifizierung und Redistribution von Zeitbudgets, den neuen Imperativen von Verlangsamung und Geduld sowie der radikal verunsicherten Zukunft, die das Virus und die wechselnden Maßnahmen zu seiner Eindämmung mit sich brachten. Andererseits kam es im Zuge der Krise zu einem Wiedererstarken des Staates als zeitpolitische Ordnungsmacht. Anstatt lediglich auf eine ordoliberale Rahmung zu setzen und die tatsächliche Ausgestaltung der zeitlichen Ordnung weitestgehend marktlichen und unternehmensinternen Koordinationsmechanismen zu überlassen, wurden Fragen von Zeitnutzung, Beschleunigung, Synchronisation, Rhythmus oder Zukunftsfähigkeit aktiv durch staatliches Handeln geprägt. Im Ausnahmezustand der Pandemie zeigt sich damit tatsächlich eine Neuordnung der Zeit und eine Rückkehr des Staates.

Die entscheidende Frage lautet jedoch, ob diese Neuordnung über den Ausnahmezustand hinaus Bestand haben kann – oder sollte. Inwiefern bietet sich im Zuge der Pandemie die Möglichkeit für eine Abkehr vom kapitalistischen Zeitregime und die Etablierung einer nachhaltigeren zeitlichen Ordnung? Während Hartmut Rosa (2020) COVID-19 als Bifurkationspunkt deutet und die staatlich durchgesetzte Entschleunigung als Chance eines nachhaltigen Pfadwechsels begreift, komme ich in meiner Betrachtung zu einem deutlich skeptischeren Resümee.

Zwar steht außer Frage, dass die Pandemie neue Erfahrungen ermöglicht und erzwungen hat, sowohl im Hinblick auf die individuelle Lebensführung als auch auf die Durchsetzungsfähigkeit des Staates. Rosa hat recht, wenn er konstatiert, dass im Zuge der Coronakrise etablierte Zeitlichkeiten infrage gestellt wurden und für manche Milieus ein neuer Umgang mit Zeit denkbar, vor allem aber auch eine Rückkehr des Staates sichtbar wurde. Die Pandemie offerierte in der Tat die Erkenntnis, dass die zeitliche Ordnung von Wirtschaft und Gesellschaft keinesfalls immun gegenüber staatlichen Eingriffen ist. Ob dies tatsächlich „eine spektakuläre politische Selbstwirksamkeitserfahrung für die Gesellschaft“ darstellt (ebd., S. 199), erscheint mir jedoch zweifelhaft.

Erstens hat die Pandemie in ihrer Kollision mit dem kapitalistischen Zeitregime nicht nur dessen unzählige Bruchstellen und Schwächen aufgezeigt, sondern eben auch die Attraktivität dieser Ordnung und die vielfältigen Abhängigkeiten von ihr verdeutlicht. Die zeitweise Abkehr vom kapitalistischen Zeitregime wurde keineswegs uniform als wohltuende Entschleunigung erlebt, wie auch Rosa (ebd., S. 207 f.) wiederholt einräumt, sondern als dramatische Erschütterung individueller und kollektiver Zeitlichkeiten, als Verlust von Freiheit, Effizienz und, ja, auch von Selbstwirksamkeit. Zeitliche Autonomie wurde in der Pandemie für viele Menschen nicht gesteigert, sondern ging in erheblichem Maße verloren. Sie wünschen sich nichts sehnlicher als ein baldiges „Nachholen“, „Aufholen“ und damit die Rückkehr zur beschleunigten Normalität.

Zweitens lässt sich die Rückkehr des Staates als zeitpolitische Ordnungsmacht und die damit einhergehenden staatlichen Eingriffe in Wirtschaft und Gesellschaft nicht pauschal als *gegen* den Kapitalismus gerichtet begreifen. Viele wirtschaftspolitische Interventionen sind eher als kurzfristiger Versuch zu verstehen, zwischen der zeitlichen Logik der nötigen staatlichen Pandemiebekämpfung und den Erfordernissen des Kapitalismus zu vermitteln. Sie taugen daher kaum als Schablone für eine nachhaltige Einhegung des kapitalistischen Zeitregimes. Insbesondere waren die verstärkten staatlichen Eingriffe kaum in der Lage, die zeitbezogenen Ungleichheiten abzubauen. Die Allokation von Zeitbudgets, Rhythmen, Geschwindigkeiten, zeitlichen Orientierungen, Reihenfolgen und zeitlicher Autonomie wurde im Zuge der Pandemie zwar weniger durch die „unsichtbare Hand des Marktes“ oder unternehmensinterne Koordinationsmechanismen bestimmt, dafür aber umso mehr durch politische Macht und Einflussnahme. Zeitbezogene Ungleichheiten wurden in der Krise daher trotz – und wegen – der Rückkehr des Staates fortgeschrieben.

Die Pandemie und die durch sie bewirkte temporäre Neuordnung der Zeit stellen einen Moment dar, in dem der gesellschaftliche Umgang mit Zeit für viele Menschen stärker ins Bewusstsein gerückt ist. Dass dieser Umstand das Potenzial für eine weitergehende Reflexion bietet, darin stimme ich mit Rosa überein. Wenn die Coronakrise aber tatsächlich als Chance für eine nachhaltigere Neuordnung der Zeit begriffen werden soll, kann die Lösung nicht in einer einfachen Rückkehr des Staates und einem Fortschreiben der zeitlichen Logik des Ausnahmezustands liegen. Stattdessen müssten die vielfältigen Dimensionen zeitbezogener Ungleichheit in ihrer Interdependenz mit dem kapitalistischen Regime in den Blick genommen werden. Nur so ließen sich staatliche Maßnahmen entwerfen, die nicht nur die marktliche und unternehmerische Koordination durch politische Entscheidungen ersetzen, sondern auch tatsächlich auf den Ausgleich zeitbezogener Ungleichheiten in kapitalistischen Gesellschaften zielen. Die Verschränkung von wirtschafts- und zeitsoziologischen Perspektiven, wie ich sie in diesem Beitrag skizziert habe, kann einen fruchtbaren Ausgangspunkt für dieses Unterfangen bieten.

## Footnotes

1 Dies bedeutet jedoch nicht, dass die Zeitregime anderer, nicht-kapitalistischer, aber moderner Gesellschaftsordnungen nicht durch ähnliche Merkmale geprägt waren bzw. sein können. So verweist die historische Forschung darauf, dass sich auch der Sozialismus durch eine starke Zukunftsorientierung, Beschleunigung und Rationalisierung von Zeit auszeichnete (Hölscher 2016; Cvetkovski 2006; Radkau 2017).

2 Vgl. dazu die klassische Abgrenzung von Arbeits- und Freizeit, beispielsweise bei Jürgen Habermas (1958).

3 Für eine kritische historische Auseinandersetzung mit dem Konzept des Ordoliberalismus als anti-liberaler Ideologie vgl. Manow (2001).

4 Die Gleichsetzung von „sozialen Kontakten“ mit der Möglichkeit zur Ansteckung ist aus soziologischer Perspektive ungenau und politisch problematisch. Wenn zwei Menschen miteinander telefonieren, sich aus der Ferne zuwinken oder in Raumfahrtanzügen gemeinsam spazieren gehen, ist ihr Handeln wechselseitig aufeinander bezogen – es handelt sich also um soziale Kontakte, aber ausdrücklich nicht um potenzielle Situationen der Ansteckung. Nutzen zwei Menschen hingegen kurz nacheinander dieselbe Zugtoilette, sehen wir keinen sozialen Kontakt, wohl aber die Gefahr einer Übertragung. Vor allem während der ersten und zweiten Welle der Pandemie wurde politisch jedoch pauschal auf die Vermeidung sozialer Kontakte und damit einhergehend auf eine gewisse soziale Passivität gedrungen, anstatt Bürger*innen zum Beispiel dazu zu ermutigen, kreativ zu werden und soziale Kontakte aktiv so zu gestalten, dass eine Übertragung von COVID-19 vermieden wird.

5 Zum Widerstreit verschiedener zeitlicher Logiken von Politik und Finanzökonomie vgl. auch Bohmann und Laux (2017).

6 Google Trends erlaubt lediglich den Zugang zu Indexwerten, die sich an den maximalen Suchanfragen im Erhebungszeitraum orientieren. Die absolute Anzahl von Suchanfragen wird genauso wenig angegeben wie Details zur Syntax der Suchanfragen. Wenngleich Google Trends mittlerweile in vielen wissenschaftlichen Disziplinen ganz selbstverständlich als Indikator eingesetzt und insbesondere für Vorhersagen und „nowcasting“ genutzt wird (Jun et al. 2018), lässt sich die Reliabilität und Validität der Daten kaum überprüfen.

7 Der Verweis auf Strategien impliziert nicht, dass die Vermittlung zeitlicher Logiken als explizites politisches Ziel benannt wurde. Die betrachteten Maßnahmen wurden öffentlich mit der Intention begründet, ökonomische Strukturen aufrechtzuerhalten und wirtschaftliche Folgeschäden zu mindern. Dass der politische Versuch, zwischen Kapitalismus und Pandemiebekämpfung zu vermitteln, auch eine zeitbezogene Komponente aufweist, wird also erst durch die wissenschaftliche zeitsoziologische Perspektive sichtbar. Unter Strategien werden hier nicht bewusste Kalküle, sondern in einem an Bourdieu (1998) orientierten praxeologischen Sinne überindividuelle Handlungsmuster verstanden, die auf den Erhalt bzw. die Herausforderung von Machtstrukturen gerichtet sind und immer auch unbewusste, den Akteuren nicht offensichtliche Komponenten enthalten.

8 Der simplen These einer Retraditionalisierung der Geschlechterverhältnisse widerspricht hingegen, dass der Anteil der Sorgearbeit, den Väter erbringen, in der Pandemie zugenommen hat.
